# Automatic classification of 3D positional relationship between mandibular third molar and inferior alveolar canal using a distance-aware network

**DOI:** 10.1186/s12903-023-03496-9

**Published:** 2023-10-25

**Authors:** So-Young Chun, Yun-Hui Kang, Su Yang, Se-Ryong Kang, Sang-Jeong Lee, Jun-Min Kim, Jo-Eun Kim, Kyung-Hoe Huh, Sam-Sun Lee, Min-Suk Heo, Won-Jin Yi

**Affiliations:** 1 Interdisciplinary Program in Bioengineering, Graduate School of Engineering, Seoul National University, Seoul, South Korea; 2 Department of Oral and Maxillofacial Radiology, Seoul National University Dental Hospital, Seoul, South Korea; 3 Department of Applied Bioengineering, Graduate School of Convergence Science and Technology, Seoul National University, Seoul, South Korea; 4 Vision AI Business Team, LG CNS, Seoul, South Korea; 5 Department of Electronics and Information Engineering, Hansung University, Seoul, South Korea; 6 Department of Oral and Maxillofacial Radiology and Dental Research Institute, School of Dentistry, Seoul National University, Seoul, South Korea

**Keywords:** Classification of 3D positional relationship, Impacted mandibular third molar, Inferior alveolar canal, Deep learning, Distance-aware network, CBCT image

## Abstract

The purpose of this study was to automatically classify the three-dimensional (3D) positional relationship between an impacted mandibular third molar (M3) and the inferior alveolar canal (MC) using a distance-aware network in cone-beam CT (CBCT) images. We developed a network consisting of cascaded stages of segmentation and classification for the buccal-lingual relationship between the M3 and the MC. The M3 and the MC were simultaneously segmented using Dense121 U-Net in the segmentation stage, and their buccal-lingual relationship was automatically classified using a 3D distance-aware network with the multichannel inputs of the original CBCT image and the signed distance map (SDM) generated from the segmentation in the classification stage. The Dense121 U-Net achieved the highest average precision of 0.87, 0.96, and 0.94 in the segmentation of the M3, the MC, and both together, respectively. The 3D distance-aware classification network of the Dense121 U-Net with the input of both the CBCT image and the SDM showed the highest performance of accuracy, sensitivity, specificity, and area under the receiver operating characteristic curve, each of which had a value of 1.00. The SDM generated from the segmentation mask significantly contributed to increasing the accuracy of the classification network. The proposed distance-aware network demonstrated high accuracy in the automatic classification of the 3D positional relationship between the M3 and the MC by learning anatomical and geometrical information from the CBCT images.

## Introduction

Extraction of the mandibular third molar (M3) is one of the most common surgeries in the oral and maxillofacial field [1–3]. Inferior alveolar nerve injury, an important surgical complication, occurs in about 0.35–8.4% of impacted M3 extractions [4]. The positional relationship between an impacted M3 and the inferior alveolar canal (MC) is the main factor that determines the risk of inferior alveolar nerve injury [5]. Panoramic radiographs have been used for preoperative imaging to predict and minimize such complications [2, 6, 7]. To predict the relationship between the M3 and the MC in panoramic radiographs, clinicians have to infer specific radiological signs (e.g., darkening or narrowing of the root, bifid apex, and interruption or diversion of the cortical outline of the MC) [8]. However, the anatomical position of the impacted M3 in relation to the MC cannot be determined easily because of superimposition and distortion of the surrounding anatomical structures in the two-dimensional (2D) panoramic radiographs [5, 9].

Cone-beam CT (CBCT) has been widely used to overcome the limitations of 2D panoramic radiographs in the oral and maxillofacial field [10]. CBCT has the advantages of lower radiation dose and cost compared with multi-detector CT, and shows three-dimensional (3D) information of anatomical structures including the teeth, jaw bone, and inferior alveolar nerve [9, 11, 12]. The 3D positional relationship between the MC and the M3 in the buccolingual direction can be determined using the cross-sectional images of CBCT. In this respect, six types of relationships were established based on the distance between the M3 and the MC, the level of contact, and the 3D positional relationship in the CBCT images [7]. The relationships were classified quantitatively based on the presence of the contact, periarticular, interradicular, buccal, and inferior positions [13]. In a previous analysis of risk factors for nerve damage with paresthesia after extraction of the M3, Wang et al. stated that the direct contact relationships between the inferior alveolar nerve and the root of the M3 as well as the buccal or lingual positions observed in preoperative CBCT images were important factors [13, 14]. Additionally, a previous study reported that the possibility of damage to the inferior alveolar nerve was higher if it was located lingually [12]. The rate of MC passage to the lingual side of the M3 root was high when the MC and the M3 were in contact [15]. Therefore, confirming the relative buccal or lingual relationship of the MC with the M3 is an essential procedure for accurate risk assessment and treatment planning to avoid or reduce inferior alveolar nerve damage during an M3 extraction.

Anatomic segmentation of the MC and M3 structures is essential when making an appropriate surgical plan based on their positional relationship to avoid or reduce nerve damage. However, segmentation of the MC and M3 and determination of the 3D positional relationship between them in relevant multiple slices of CBCT images is time-consuming and labor-intensive. Automatic segmentation methods have been proposed including level-set methods [16–20], template-based fitting methods [21], and statistical shape models [22, 23]. However, these methods have some limitations, such as initialization problems, transformation vulnerability, and additional manual annotation, which need improvement for fully automatic segmentation. Recently, many studies based on deep learning have been performed on the segmentation and classification of anatomical structures or lesions in medical or dental images [24–26], and they have shown impressive performance improvement in terms of overcoming limitations [27–33]. Several studies for detecting and segmenting the MC in CBCT images have also been performed using deep learning [27, 29]. The proximity and contact relationship between the M3 and the MC were classified in CBCT images using a ResNet-based deep learning model [34]. To the best of our knowledge, no previous studies have attempted to apply deep learning to the classification of the relative buccal or lingual relationships between the M3 and the MC. Thus, in this study, we focused on developing an end-to-end automatic method based on deep learning to replace the time- and labor-consuming process of determining the relative buccal or lingual relationship between the M3 and the MC.

We hypothesized that a deep learning model could automatically determine the relative buccal or lingual relationship between the M3 and the MC using distance information in CBCT images. Therefore, the purpose of this study was to automatically classify the 3D positional relationship between an impacted M3 and the MC using a 3D distance-aware network that consisted of cascaded stages of segmentation and classification of CBCT images. Our main contributions were that we proposed a distance-aware network for automatic and accurate classification of the 3D positional relationship between the M3 and the MC by learning anatomical and geometrical information. In addition, we applied the signed distance map (SDM) generated from the segmentation mask as a multichannel volumetric input in the 3D distance-aware classification network to guide the position and distance relationships between the M3 and the MC.

## Materials and methods

### Data acquisition and preparation

This study was performed with approval from the Institutional Review Board (IRB) of Seoul National University Dental Hospital (ERI18001). The ethics committee approved the waiver for informed consent because this was a retrospective study. The study was performed in accordance with the Declaration of Helsinki. CBCT images were obtained from 50 patients (27 females and 23 males; mean age 25.56 ± 6.73 years) who underwent dental implant surgery or extraction of the M3 at Seoul National University Dental Hospital in 2019–2020. The images had dimensions of 841 × 841 × 289 pixels, voxel sizes of 0.2 × 0.2 × 0.2 mm^3^, and 16-bit depth and were obtained at 80 kVp and 8 mA using a CBCT (CS9300; Carestream Health, New York, USA).

The anatomical structures of the M3 and the MC in the CBCT images were manually annotated by an oral and maxillofacial radiologist using the 3D Slicer for Windows 10 (Version 4.10.2; MIT, Massachusetts, USA) [35]. The ground truth of the MC was established by annotation of both the inferior alveolar nerve and the cortical bone. For multiclass segmentation of the M3 and the MC by deep learning, we prepared 64 volumes (32 patients) for the training dataset and 36 volumes (18 patients) for the test dataset from all CBCT images (50 patients), where the right mandible volume was horizontally flipped to match the left (Table 1). The training and test datasets used for segmentation comprised 3546 and 1804 axial images, respectively (Table 1). The 2D axial images were automatically cropped into images of 512 × 512 pixels centered at the region of the mandible as an input of the segmentation network.

**Table 1:** The dataset configuration of CBCT images used for deep learning

	Patients	Volumes	Axial sections	Buccal cases	Lingual cases
Train, Validation	32	64	3546	43	8
Test	18	36	1804	20	11
Total	50	100	5350	63	19

**Fig. 1:**
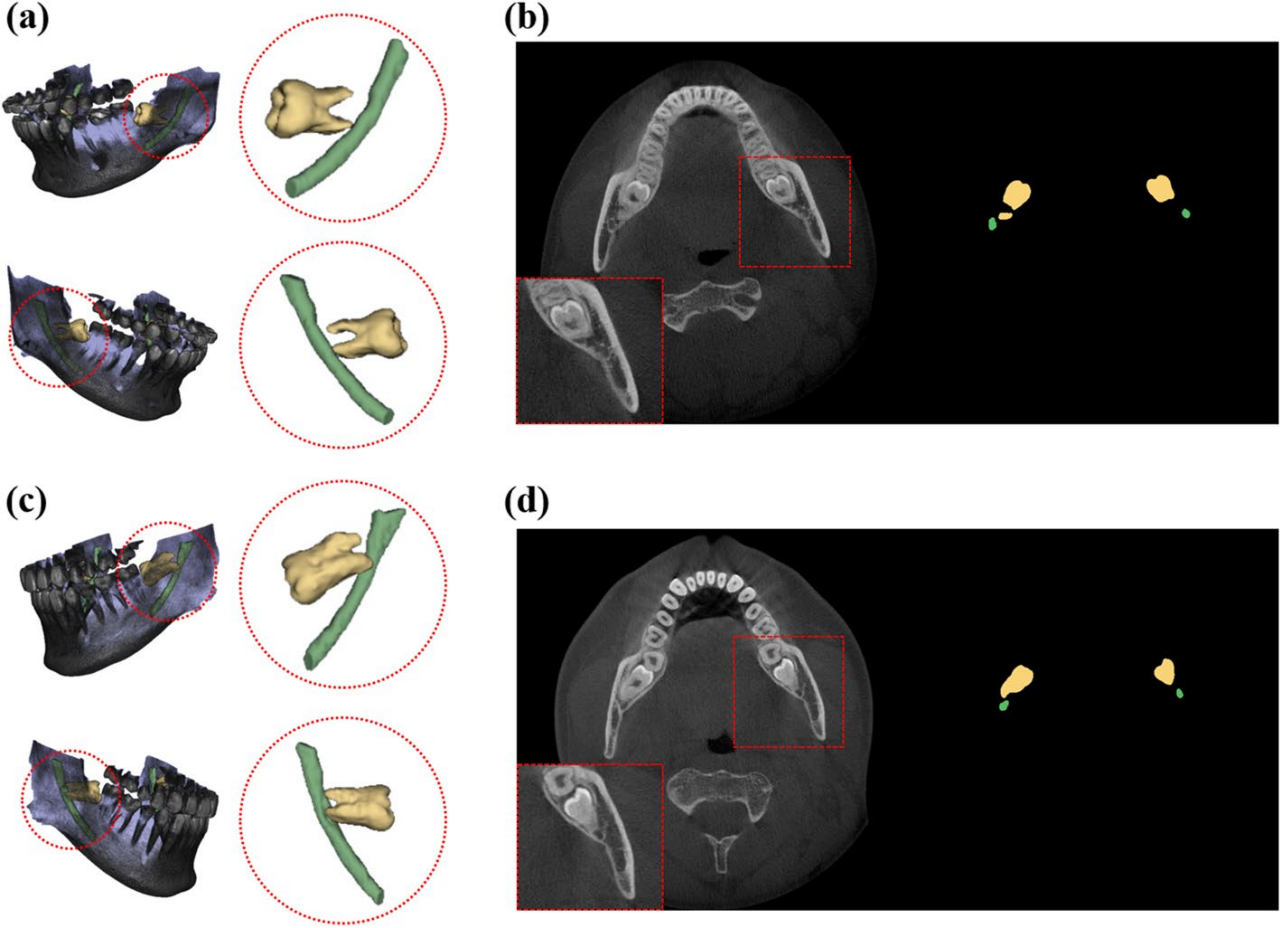
Visualization of mandibular canals running along the buccal side of the impacted mandibular third molar (**a**, **b**) and of mandibular canals running along the lingual side of the impacted mandibular third molar (**c**, **d**) in 3D and 2D axial slices of a CBCT image and the ground truth of segmentation

The buccolingual relationship of the M3 and the MC was determined by analysis of successive slices in multiplanar CBCT images by an oral and maxillofacial radiologist. The passing direction and path of the MC were evaluated based on the lamina dura of the M3. If the MC directly contacted or passed in close proximity to the inner surface of the M3 root, it was considered a lingual class, but if it directly contacted or passed in close proximity to the outer surface of the M3 root, it was classified as a buccal class (Fig. 1). We excluded the CBCT images in which it was difficult to determine the positional relationship between the MC and M3 for classification dataset. In our study, the radiologist annotating the images was unaware of critical information that could bias their assessments. This information included the patient's dental history, the patient's clinical symptoms, or demographic information. When the interpreter was not aware of the patient's clinical information, they were less likely to make assumptions about the patient's condition based on that information. This could lead to a more accurate interpretation of the image.

For classification by deep learning, we prepared 43 buccal and 8 lingual cases for the training dataset and 20 buccal and 11 lingual cases for the test dataset from the whole dataset of 63 buccal and 19 lingual cases. The CBCT images for the input of the classification network were cropped from the segmentation results into a volume of 256 × 256 × 32 pixels. We estimated the minimum required sample size to detect significant differences in the accuracy between the distance-aware network and the other networks when both assessed the same subjects. Based on an effect size of 0.50, a significance level of 0.05, and a statistical power of 0.80, we obtained a sample size of *N* = 128 (G* Power for Windows 10, Version 3.1.9.7; Universität Düsseldorf, Germany). Therefore, we split all CBCT images into 3546 and 1804 axial images for the training and test datasets, respectively.

### Overall architecture of the distance-aware network

**Fig. 2:**
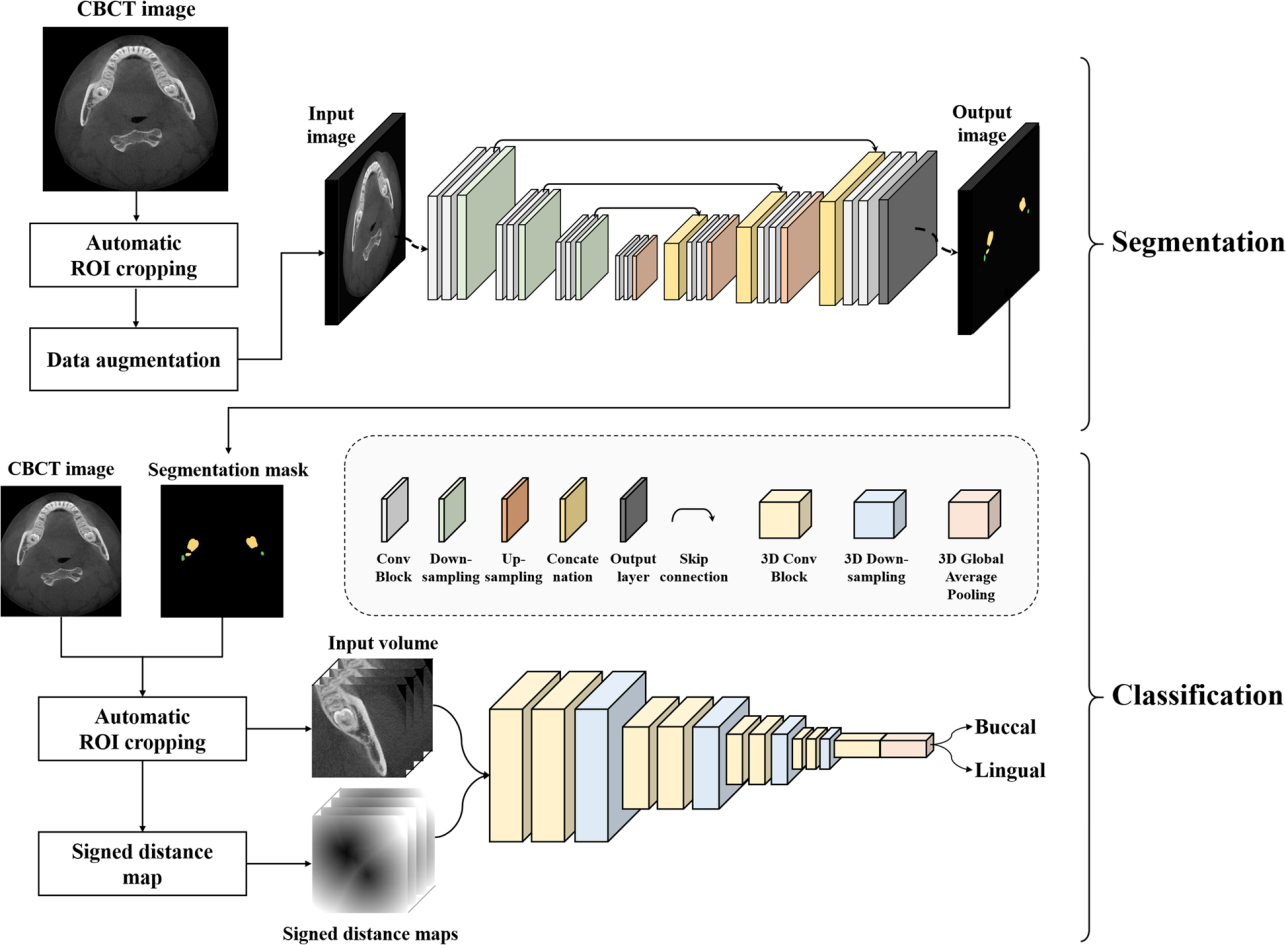
The 3D distance-aware network consisting of segmentation and classification stages for classifying the positional relationship between the third molar and the mandibular canal. In the segmentation stage, the third molar and the mandibular canal were simultaneously segmented using Dense121 U-Net. In the classification stage, the 3D distance-aware network with inputs of CBCT volume and the distance map classified the buccal-lingual positional relationship between the third molar and the mandibular canal

We designed a distance-aware network consisting of cascaded stages of segmentation and classification (Fig. 2). In the segmentation stage, a U-Net of DenseNet121 [36] backbone with the input of 2D axial images (S-Net) was used for multiclass segmentation of the M3 and the MC, simultaneously. In the classification stage, a 3D distance-aware classification network with input of multiple volumes (C-Net) was designed for classifying the buccal-lingual relationship of the M3 and the MC by learning their 3D anatomical and geometrical information.

For multiclass segmentation of the M3 and the MC, we used a pre-trained DenseNet121 backbone as the encoder of the U-Net, which consisted of multiple densely connected layers and transition layers to improve feature propagation and alleviate the vanishing gradient problem (Fig. 2). The decoder was composed of a five-level structure where each level consisted of a 2 × 2 up-sampling layer, a skip connection, and two convolutional blocks. Each convolutional block consisted of a 3 × 3 convolutional filter, a batch-normalization layer, and a rectified linear unit (ReLU) activation function. The SoftMax activation function was applied to the last activation layer for outputting multiclass segmentation of both the M3 and the MC. Several U-shaped networks such as SegNet [37], simple U-Net [38], and Attention U-Net [39] were also used as segmentation networks for performance comparison.

For classification of the positional relationship between the M3 and the MC, we designed a 3D distance-aware network (C-Net) consisting of a five-level structure where each level consisted of a 3 × 3 × 3 convolutional layer, a batch-normalization layer, the ReLU activation function, and a 2 × 2 × 2 max-pooling layer (Fig. 2). The original CBCT volume, binary segmentation mask from predictions, and corresponding SDM were used as multichannel inputs for the C-Net. The input volumes were centered at the point where the M3 and the MC were closest. In the output layer, the class probability for the relative buccal-lingual position of the MC was calculated using the SoftMax activation function following the global average pooling layer and the dense layer. The predictions by the segmentation networks of SegNet, simple U-Net, Attention U-Net, and Dense U-Net were also used as the input of the classification network for performance comparison.

The segmentation networks were trained using the Dice similarity coefficient loss and the Adam optimizer with a learning rate of 0.00025 that was reduced on a plateau by a factor of 0.5 every 25 epochs over 300 epochs with a batch size of 8. The classification network was trained using binary cross-entropy and the Adam optimizer with a learning rate of 0.001 that was reduced on a plateau by a factor of 0.5 every 25 epochs over 100 epochs with a batch size of 1. Analyses were implemented with Python3 based on Keras with a Tensorflow backend using a single NVIDIA Titan RTX GPU 24 GB.

#### Signed distance map (SDM) for positional information

**Fig. 3:**
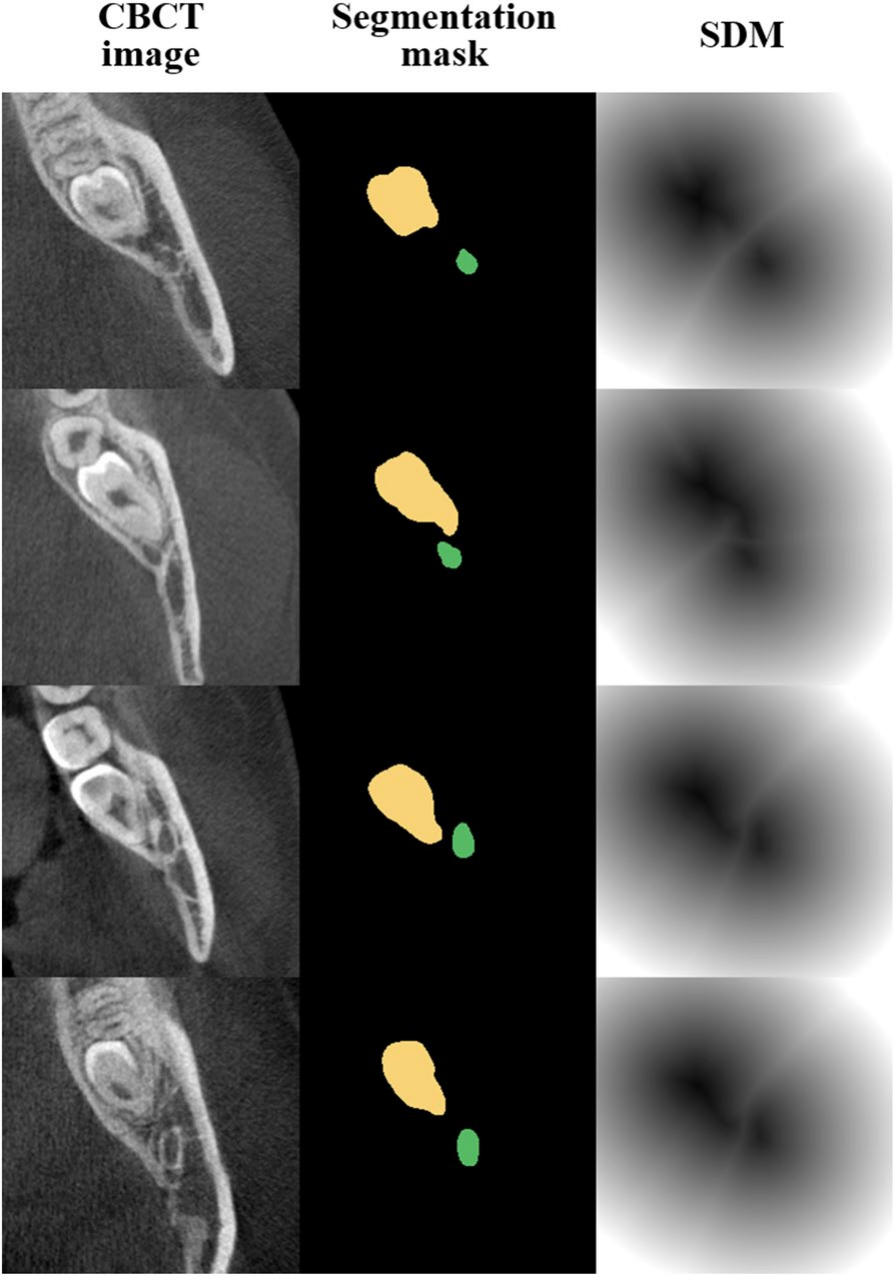
Examples of axial image (left), segmentation mask (middle), and SDM (right) of the mandibular third molar and the canal. The demarcation line was observed radially in the signed distance map, and the difference in direction was observed when the mandibular canals traveled along the lingual side or buccal side of the M3

In the classification process, the network learned the 3D anatomical and geometrical information from the multichannel volume inputs simultaneously. The SDM calculated from the mask result of the segmentation prediction was used as an input for learning the geometrical information, while the original CBCT image was used for learning the 3D anatomical information (Fig. 3). The geometric SDM between the M3 and the MC from the segmentation prediction was calculated as the signed distance transform (SDT) [40]. The SDT was defined as the Euclidean distance from the nearest background point:

1 $$SDT\left(x\right)= \left\{\begin{array}{c}d\left(x,\partial M\right) if x\in M\\ -d\left(x,\partial M\right) if x\in {M}^{c}\end{array}\right.$$

where $x$ is the metric space, $M$ is the metric space of the M3 and the MC, and $\partial M$ is the boundary of $M$ [41, 42]. For any $\in X,$ $\left(x,\partial M\right):=\underset{y\in \partial M}{\mathrm{inf}}d(x,y)$, where *inf* denotes the infimum.

The SDM was derived from the 3D SDT considering the internal shape of the object and the external relationship (Eq. 2), where *B* denoted the binary segmentation mask of the M3 and the MC. When calculating the SDM, the inside and outside of a boundary of an object had a negative and a positive value, respectively. In the SDM from the predicted M3 and MC segmentations, the values between the boundaries of the two objects formed a line that had a constant value. This information helped the networks to learn the geometrical relationship between the M3 and the MC.

2 $$SDM=\left(1-B\right)*SDT\left(1-B\right)-B*(SDT\left(B\right)-1)$$

where $B$ denotes the binary segmentation mask of the M3 and the MC.

#### Performance evaluation for segmentation and classification

The decision-making process of the networks for buccolingual classification was visualized and verified in the attentional area of the classification networks with multichannel inputs using gradient-weighted class activation mappings (Grad-CAMs). Grad-CAM is a powerful tool for interpreting the decision-making process of a deep learning network by visualizing the learning results of the network in terms of heat maps [43]. Therefore, a qualitative evaluation was performed by confirming the differences in the attentional areas of the Grad-CAM among the classification methods based on the segmentation network and multichannel inputs.

The segmentation performance of the Dense121 U-Net was compared with those of other networks from SegNet [37], simple U-Net [38], and Attention U-Net [39]. The evaluation matrix included precision, recall, Dice similarity coefficient (DSC), intersection over union (IoU), 3D volumetric overlap error (VOE), and relative volume difference (RVD). All matrices were calculated as volume levels. Precision $(\frac{TP}{TP+FP}$) was the rate of correctly predicted positive predictions, recall $(\frac{TP}{TP+FN}$) was the rate of correctly predicted ground truths, and DSC $(\frac{2TP}{2TP+FN+FP})$ was a harmonic mean of precision and recall, where TP, FP, and FN denoted true positive, false positive, and false negative, respectively. VOE $(1- \frac{{V}_{gt}\cap {V}_{pred}}{{V}_{gt}\cup {V}_{pred}}$) is the rate between intersection and union of two sets of segmentations, and RVD ($\frac{|{V}_{gt}-{V}_{pred}|}{{V}_{gt}}$) is the absolute volumetric size difference of the regions, where ${V}_{gt}$ and ${V}_{pred}$ represent the number of voxels for the ground truth and the predicted volumes, respectively. Higher values of DSC, precision, recall, and IoU and lower values of VOE and RVD indicate better segmentation performance. The precision-recall curve was also computed from the network’s segmentation output by varying the IoU threshold. Average precision (AP) was calculated as that across all recall values.

The classification performance of the Dense121 U-Net with a variety of configurations of volume inputs was compared with other networks with the same volume inputs. The classification performance was evaluated using sensitivity, specificity, accuracy, and the area under the receiver operating characteristic curve (AUC). Sensitivity $(\frac{TP}{TP+FN}$) correctly identified the positive result for the actual class, specificity $(\frac{TN}{TN+FP}$) correctly identified the negative result for the actual class, and accuracy $(\frac{TP+TN}{TP+TN+FP+FN}$) provided the proportion of correct predictions for all classes, where TP, FP, TN, and FN denoted true positive, false positive, true negative, and false negative, respectively. The receiver operating characteristic curve (ROC) was also computed from the network classification output by varying the class probability for each network.

## Results

Table 2 shows the quantitative results of the segmentation performance of IoU, DSC, precision, recall, RVD, and VOE by U-Net models. The performance of Dense121 U-Net, Attention U-Net, Simple U-Net, and SegNet was compared over the 36 volumes of the test dataset. The Dense121 U-Net achieved segmentation performances of IoU, DSC of precision, recall, RVD, and VOE of 0.872, 0.920, 0.946, 0.918, 0.038, and 0.088, respectively, for the M3, and of 0.766, 0.861, 0.911, 0.830, 0.135, and 0.248, respectively, for the MC. The Attention U-Net showed similar or better performance in some parameters. Generally, the Dense121 U-Net showed the highest scores in the segmentation performances for the M3 and the MC among the U-Net models.

**Table 2:** Segmentation performances (Mean (SD)) of intersection over union (IoU), Dice similarity coefficient score (DSC), precision (PR), recall (RC), relative volume difference (RVD), and volume of error (VOE) by Dense121 U-Net, Attention U-Net, simple U-Net, and SegNet for the mandibular third molar and the mandibular canal

**Mandibular third molar**						
Segmentation models	IoU	DSC	Precision	Recall	RVD	VOE
SegNet	0.841 ± 0.183	0.898 ± 0.156	0.939 ± 0.093	0.888 ± 0.176	0.049 ± 0.031	0.111 ± 0.027
simple U-Net	0.846 ± 0.181	0.902 ± 0.147	0.944 ± 0.092	0.889 ± 0.177	0.056 ± 0.034	0.105 ± 0.034
Attention U-Net	0.863 ± 0.161	0.916 ± 0.125	0.940 ± 0.086	0.911 ± 0.151	0.038 ± 0.021	0.093 ± 0.027
Dense121 U-Net	0.872 ± 0.161	0.920 ± 0.131	0.946 ± 0.091	0.918 ± 0.148	0.038 ± 0.025	0.088 ± 0.024
**Mandibular canal**						
Segmentation models	IoU	DSC	Precision	Recall	RVD	VOE
SegNet	0.659 ± 0.212	0.770 ± 0.194	0.837 ± 0.171	0.741 ± 0.226	0.162 ± 0.104	0.357 ± 0.146
simple U-Net	0.721 ± 0.162	0.825 ± 0.133	0.899 ± 0.106	0.785 ± 0.176	0.192 ± 0.106	0.303 ± 0.114
Attention U-Net	0.770 ± 0.132	0.863 ± 0.102	0.898 ± 0.093	0.846 ± 0.143	0.131 ± 0.074	0.249 ± 0.077
Dense121 U-Net	0.766 ± 0.125	0.861 ± 0.096	0.911 ± 0.085	0.830 ± 0.136	0.135 ± 0.071	0.248 ± 0.075

**Fig. 4:**
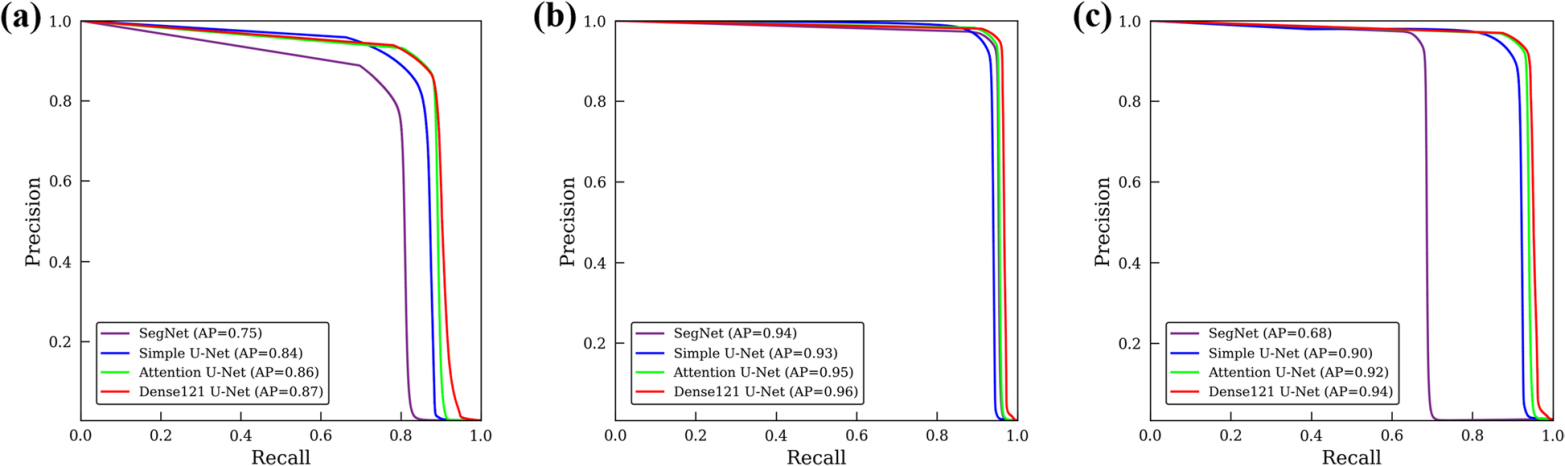
Precision-recall curves from segmentation results for M3 (**a**), MC (**b**), and both together (**c**)

**Fig. 5:**
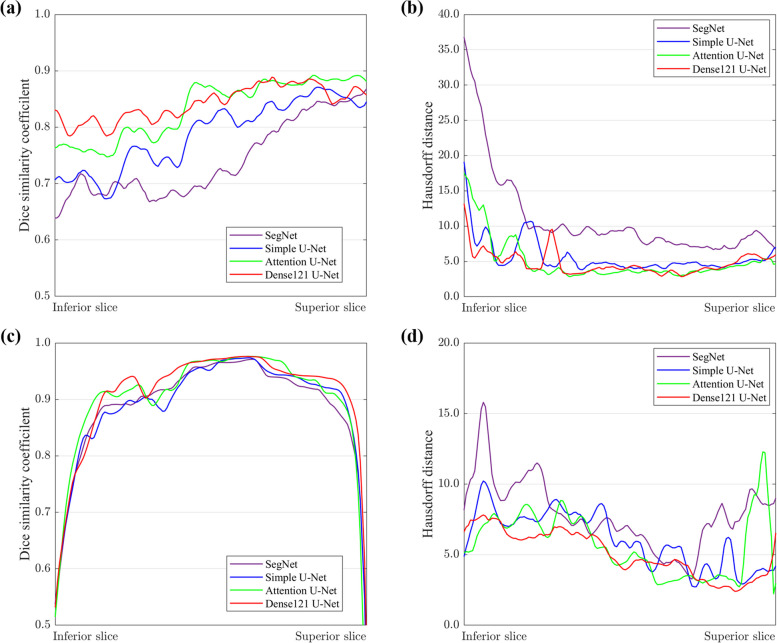
The line plots of Dice similarity coefficient (**a**, **c**) and Hausdorff distance (**b**, **d**) by Dense121 U-Net, Attention U-Net, simple U-Net, and SegNet for the M3 (**a**, **b**) and MC (**c**, **d**). The metric values were calculated from the inferior axial slice to the superior slice in the volume

Figure 4 presents the precision-recall curves of the segmentation performance for the M3 and the MC by SegNet, simple U-Net, Attention U-Net, and Dense121 U-Net. Of the tested networks, the Dense121 U-Net achieved the highest AP of 0.87, 0.96, and 0.94 for the M3, the MC, and both combined, respectively (Fig. 4). Figure 5 shows the line plots for the means of DSC and Hausdorff distance values calculated from the inferior axial slice to the superior one for the M3 and the MC volumes by the U-Net models. The Dense121 U-Net usually predicted the M3 and the MC volumes more accurately with smaller fluctuations in performance.

**Fig. 6:**
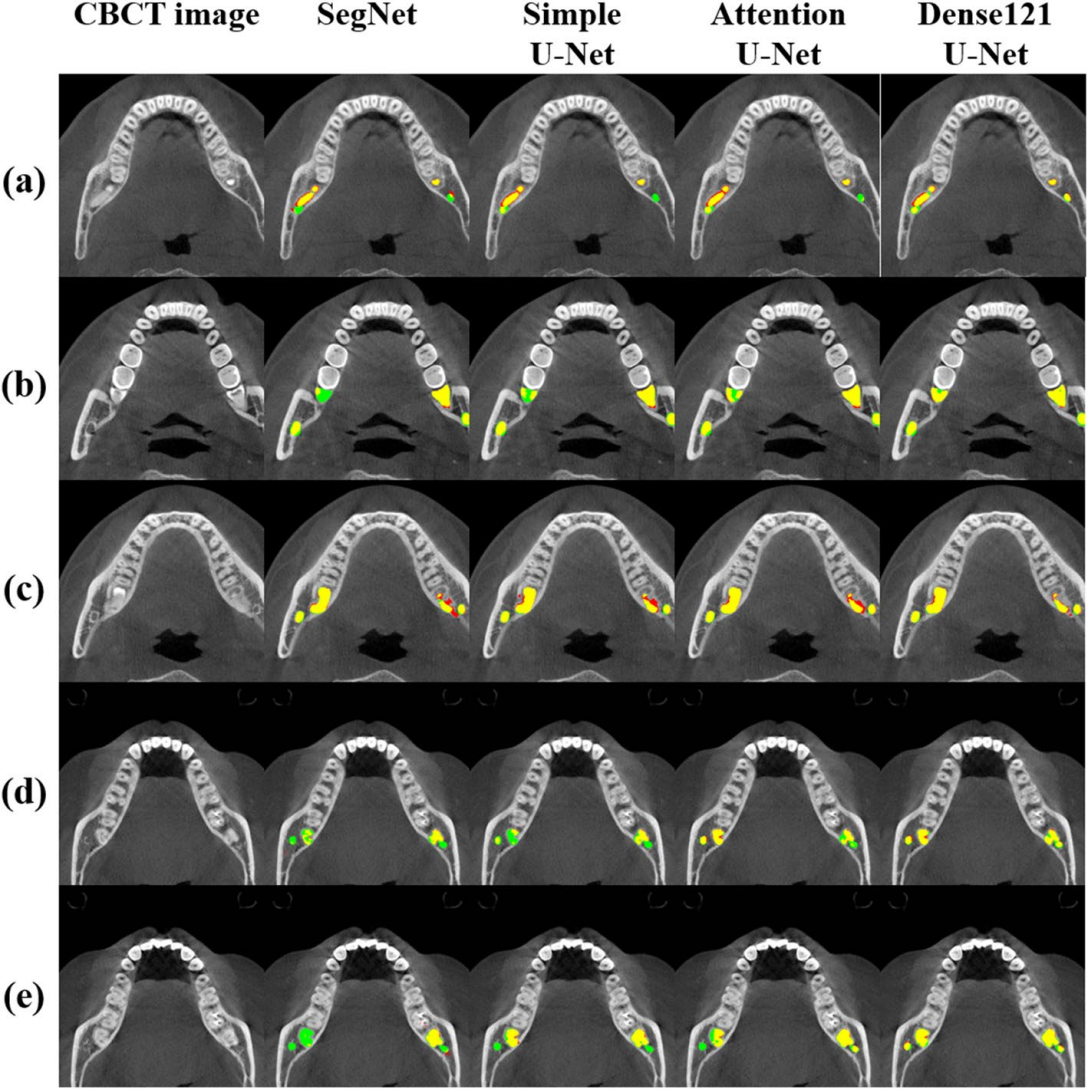
Segmentation results for the M3 and the MC by SegNet, simple U-Net, Attention U-Net, and Dense121 U-Net. The predicted segmentation masks of the M3 and the MC were superimposed on the ground truth in CBCT images. The yellow, green, and red regions represent true positive, false negative, and false positive, respectively

**Fig. 7:**
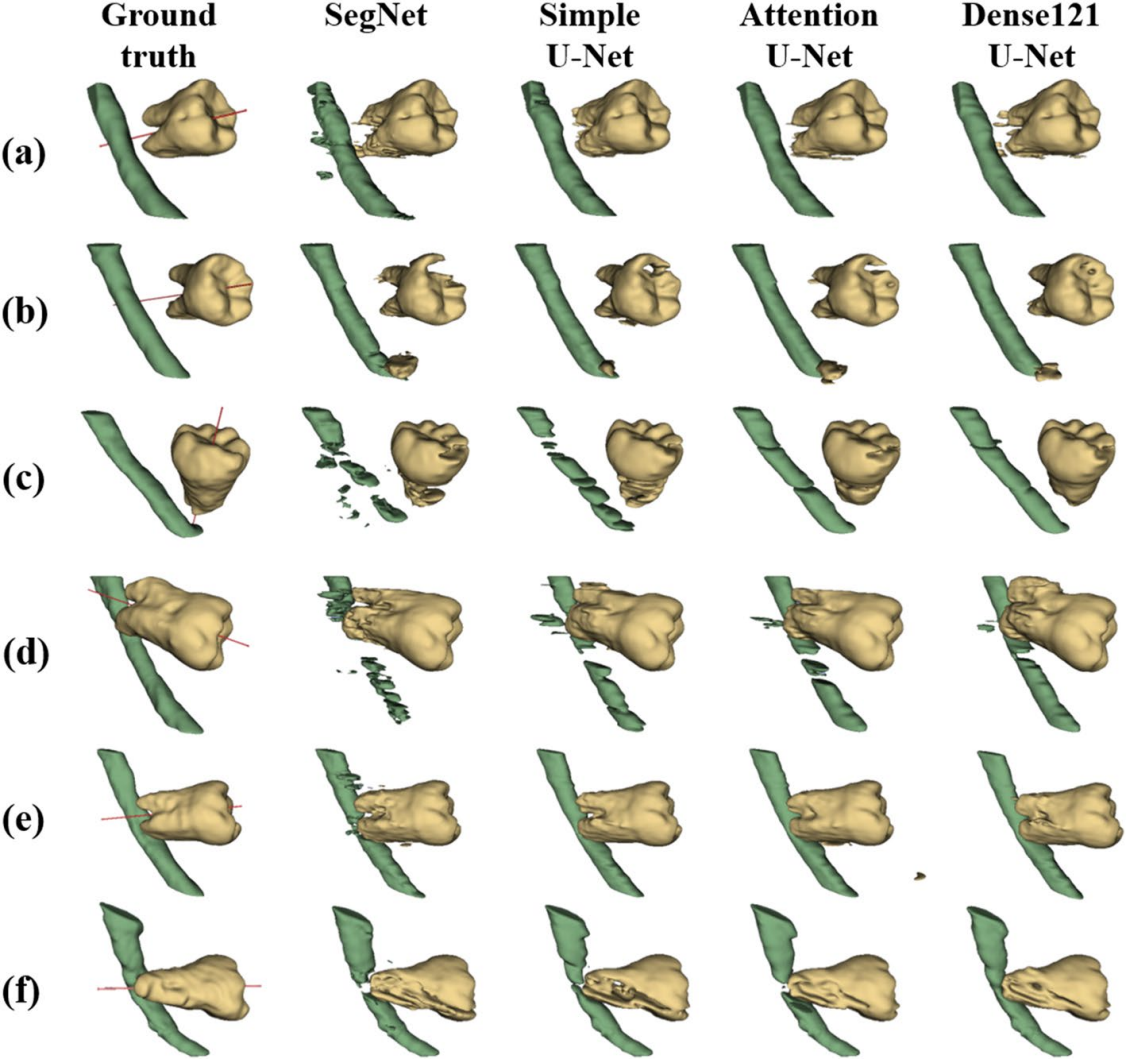
The 3D visualization of the ground truth and segmentation results from SegNet, simple U-Net, Attention U-Net, and Dense121 U-Net from left to right with buccal relationships between M3 and MC (**a**-**c**) and with lingual relationships (**d**-**f**). The red line passing through the M3, the main axis of the M3, shows the buccal-lingual relationship between the M3 and the MC on the ground truth

Figures 6 and 7 present the segmentation results for the M3 and the MC in 2D and 3D by the U-Net models. The predicted segmentations for the M3 and the MC by the networks and the ground truth are superimposed on the axial CBCT image (Fig. 6). The 2D segmentation results from the Dense121 U-Net show more true positives (yellow) and fewer false negatives (green) and false positives (red) in the MC and M3 areas compared to the other networks (Fig. 6). Particularly, the Dense121 U-Net successfully segmented the areas where the cortical bone of the MC was not clearly visible due to compression or obstruction by the root of the M3, while the other networks failed to segment these areas in the CBCT images (Fig. 6c–e). As a result, 3D segmentation by Dense121 U-Net exhibited better prediction results with improved structural continuity and boundary details for the MC and M3 volumes compared with the other networks (Fig. 7).

**Fig. 8:**
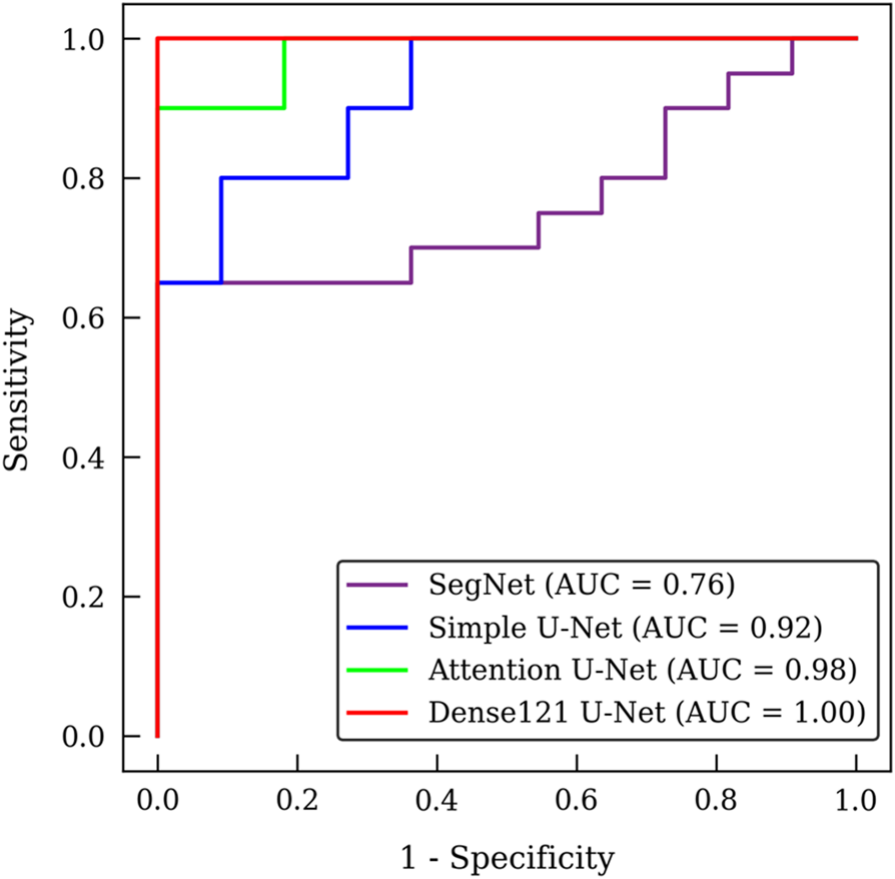
The classification performance of the 3D distance-aware networks based on the receiver operating characteristic (ROC) curves. The ROC curves and AUCs from the networks with segmentation networks of SegNet, simple U-Net, Attention U-Net, and Dense121 U-Net

The results in Table 3 show the classification performances for the buccal-lingual relationship between the M3 and the MC by the 3D distance-aware networks with different configurations of input volumes from the original CBCT volume and the binary segmentation mask and SDM from Dense121 U-Net, Attention U-Net, simple U-Net, and SegNet. The classification performances by the 3D classification networks with the input of the original CBCT volume and/or the segmentation mask showed lower accuracy, sensitivity, specificity, and AUC. In contrast, higher classification performances were achieved by the 3D classification networks with the input of the SDM volume. The 3D classification network by Dense121 U-Net with the input of both the CBCT image and the SDM showed the highest performance, with accuracy, sensitivity, specificity, and AUC all having values of 1.00, whereas Attention U-Net had accuracy, sensitivity, specificity, and AUC values of 0.90, 0.90, 0.91, and 0.98, respectively. The ROC curve of classification performance by Dense121 U-Net and Attention U-Net using the CBCT image and the SDM is closest to the upper left, with AUC values of 1.00 and 0.98, respectively (Fig. 8). A lower segmentation accuracy of the segmentation networks represented with SDM caused by discontinuous, fragmented, and scattered structures led to a lower classification accuracy of the 3D distance aware networks. This showed difficulty in learning the proper relationships between the M3 and the MC. The classification performance was influenced by the performance of the segmentation network as the SDM input of the classification network was directly derived from the segmentation mask.

**Table 3:** Classification performances of accuracy, sensitivity, specificity, and area under the receiver operating characteristic curve (AUC) by the 3D distance-aware networks with different configurations of input volumes of the original CBCT image, segmentation mask, and SDM by Dense121 U-Net, Attention U-Net, simple U-Net, and SegNet. SDM: signed distance map

Segmentation Model	CBCT image	Segmentation mask	SDM	Accuracy	Sensitivity	Specificity	AUC
	✔			0.32	0.10	0.73	0.34
SegNet	✔	✔		0.48	0.25	0.91	0.69
simple U-Net	✔	✔		0.77	0.80	0.73	0.83
Attention U-Net	✔	✔		0.52	0.40	0.73	0.62
Dense U-Net	✔	✔		0.71	1.00	0.18	0.69
SegNet			✔	0.77	0.65	1.00	0.91
simple U-Net			✔	0.84	0.80	0.91	0.96
Attention U-Net			✔	0.71	0.85	0.45	0.85
Dense U-Net			✔	0.84	0.90	0.73	0.92
SegNet	✔		✔	0.65	0.65	0.64	0.76
simple U-Net	✔		✔	0.68	1.00	0.09	0.92
Attention U-Net	✔		✔	0.90	0.90	0.91	0.98
Dense U-Net	✔		✔	1.00	1.00	1.00	1.00

**Fig. 9:**
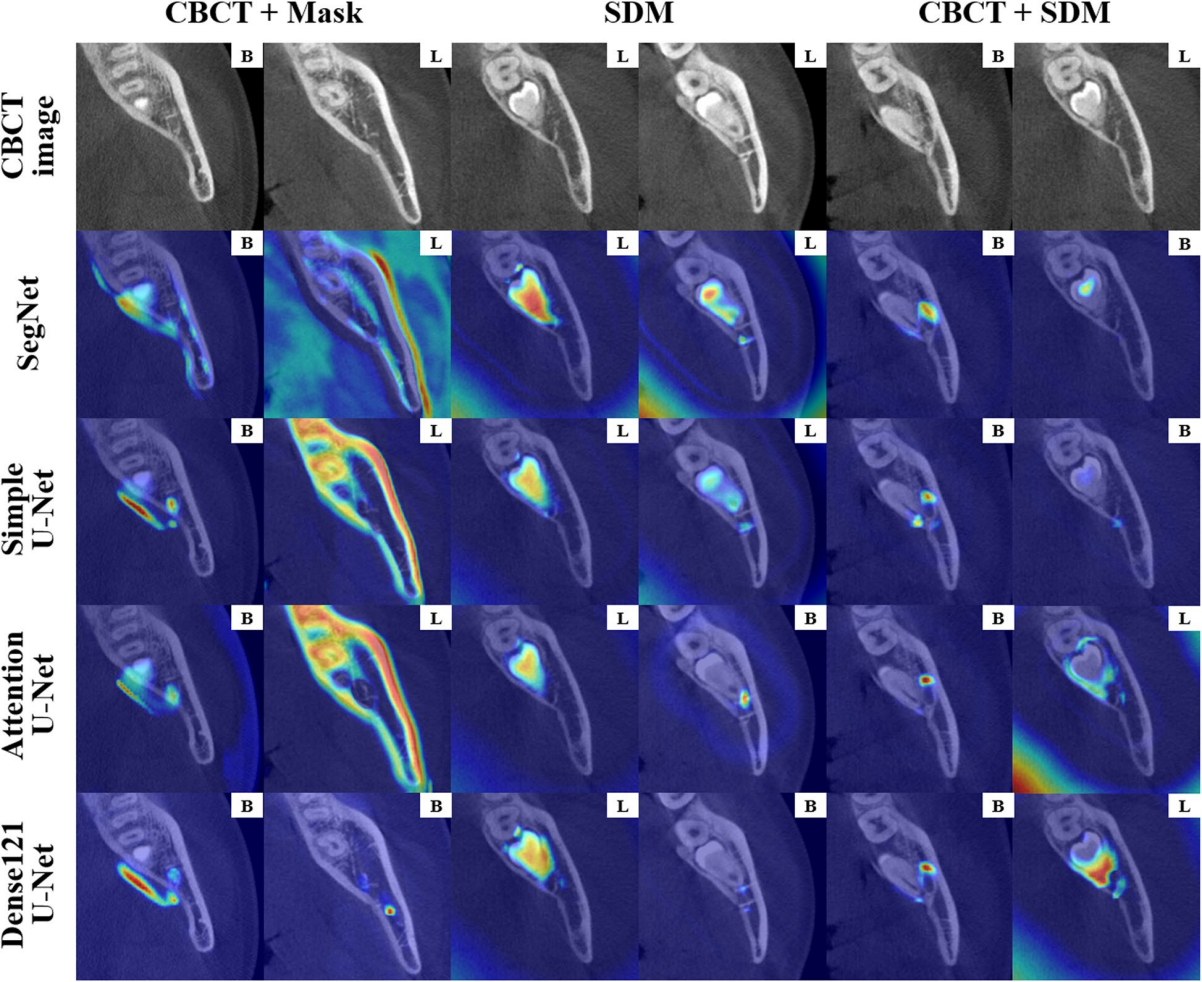
Visualization of Grad-CAM results from the 3D distance-aware networks by SegNet, simple U-Net, Attention U-Net, and Dense121 U-Net with inputs of the CBCT image and segmentation mask (left), the SDM (middle), and the CBCT image and SDM (right). Ground truth (on CBCT image) and predicted classes are shown on each image, where B and L indicate buccal and lingual relationships, respectively

Figure 9 shows activation heatmaps of the Grad-CAM superimposed on the CBCT images according to the segmentation networks and the input configuration of the classification network. The classification networks using the inputs of the original CBCT image and segmentation mask did not emphasize properly the anatomical regions related to the classification because the networks identified the entire mandible and M3 or external regions that were not relevant to the classification. However, the classification networks using the inputs of the CBCT image and SDM returned reasonable areas for classification. The areas of the M3 root and the medullary space surrounding the cortical layer of the MC were identified well in cases where the network predicted the lingual relationship, and the marginal area of the M3 and the medullary space near the cortical layer of the MC was identified well in cases with a buccal relationship.

## Discussion

Accurate segmentation of the inferior alveolar nerve is an essential step in oral and maxillofacial surgery, such as implant placement in the mandible, extraction of an impacted M3, and orthognathic surgery [1–3, 44]. The incidence of inferior alveolar nerve injuries in M3 extraction surgery increases if the MC and the root of the M3 are closely located [4]. In addition, the positional relationship of the MC with the M3 in the buccolingual aspect is a risk factor as important as proximity and is identified routinely in the clinical field [14, 45]. Therefore, diagnosis based on preoperative imaging is essential for accurate prediction of and sufficient preparation for this risk. CBCT images, which overcome the limitations of distortion and superimposition of structures in 2D panoramic images [5, 9], have been widely used in dental clinics [10]. However, accurate identification of the path of the MC and its 3D relationship with the M3 on cross-sections of the CBCT image is labor- and time-consuming due to the low contrast and high noise [46].

The application of deep learning for segmentation or classification of the M3 or the MC has been performed in 2D panoramic radiographs, 3D CT, and CBCT images [27, 29, 31, 47–50]. Another study has segmented the M3 and the MC in CBCT images and classified them into three types according to proximity and contact between the two structures [34]. However, no previous studies have been performed on the automatic classification of the buccal-lingual positional relationship between the M3 and the MC using deep learning. In this study, a distance-aware CNN was proposed to segment the M3 and the MC and to classify the positional relationship between them in CBCT images, automatically. In the segmentation stage, pre-trained Dense121 U-Net was used for multiclass segmentation of the M3 and the MC. In the classification stage, a 3D classification network simultaneously learned anatomical and geometrical information from the CBCT image and the distance map generated from the segmentation prediction of the M3 and the MC.

The M3 and MC segmentation by Dense121 U-Net showed the highest performance among all segmentation networks, indicating that the network had balanced precision and recall. The results of the Dense121 U-Net showed a significant reduction in the number of discontinuous areas where the cortical bone of the MC was not clearly visible and was compressed or obstructed by the root of the M3 in CBCT images. Those of other networks showed a larger number of areas of discontinuity for the MC. The plots representing the segmentation performance of DSC and Hausdorff distances for the M3 and the MC by Dense121 U-Net demonstrated this quantitatively with higher values and smaller fluctuations compared to those of the other networks. These results showed that the Dense121 U-Net is the most effective network for segmenting small target objects with consistent accuracy. Therefore, 3D segmentation by Dense121 U-Net demonstrated the best performance with improved structural continuity and boundary details for segmentation of the MC and the M3.

The 3D distance-aware network was designed to automatically classify the buccal-lingual relationship between the M3 and the MC, and it incorporated the SDM as an input to learn additional 3D geometrical information between them. Using both the 3D CBCT image and the SDM as multichannel inputs of the network, the network achieved better classification performance compared to other input combinations. The performance of the classification network was highest, reaching 0.90 or more when using an SDM generated from the segmentation outcomes of Attention U-Net or Dense121 U-Net with higher segmentation performance. These results demonstrated that the classification network using both the original CBCT image and the SDM as multichannel inputs learned the 3D positional information more effectively to analyze the relationship between the M3 and the MC.

We utilized Grad-CAM visualization to create visual interpretations for the decision-making process in the buccolingual classification of the M3 and the MC by the classification network. The heat maps generated by the networks using inputs of the CBCT image and SDM focused specifically on the root of the M3 and the periphery of the MC, while those using other inputs emphasized regions that were not relevant to classification. As a result, the SDM provided geometrical guidance that provided precise information on the position and distance relationships between the M3 and the MC during classification. In addition to the information on the anatomical regions for the M3 and the MC, the geometrical information for the position and shape of adjacent anatomical structures was important for better classification performance.

The purpose of including a control group in a study is to provide a baseline for comparison. In studies like ours, the control group can be used to compare the results of the group which uses the developed network in classification for the buccal-lingual relationship in order to see if there is a statistically significant difference. As there was no control group in this study, it was difficult to say whether the group using the developed network demonstrated better performance in the classification than the control group not using the network. However, deep learning models in medical and dental image interpretation can be advantageous in the following ways [24, 30, 51, 52]. Deep learning models can interpret medical images much faster than human interpreters, which can lead to a faster diagnosis for patients [30]. Deep learning models can automate the process of medical image interpretation, which can free up human interpreters to focus on other tasks [30, 51]. Deep learning models can help to improve the efficiency of medical image interpretation by reducing the time and labor required [24, 52].

Our study did have some limitations. First, due to the insufficient availability of the CBCT images, only the relative buccal or lingual relationship between the M3 and the MC was considered as a criterion for classification, although the MC could appear in a greater variety of positions and orientations with respect to the M3, such as through, beneath, or between the roots. Future studies should include more diverse criteria for detailed classification. Second, our study had a potential limitation of generalization ability due to the use of CBCT images from a single organization. Overfitting of training of a deep learning model, which results in the model learning statistical regularity specific to the training dataset, could negatively impact the ability of the model to generalize to a new dataset [53]. Therefore, in future studies, it is important to improve and evaluate the performance of the network on CBCT images of a variety of relationships of the M3 and the MC and those obtained from different devices.

## Conclusions

In this study, the proposed distance-aware network demonstrated high accuracy in automatic classification of the 3D positional relationship between the M3 and the MC by learning anatomical and geometrical information from CBCT images. The distance map generated using the segmentation mask as one of the multichannel inputs in the classification network significantly contributed to increasing the accuracy in the classification of the 3D buccal-lingual relationship between the M3 and the MC. The network could contribute to the automatic, accurate, and efficient classification of the 3D positional relationship between the M3 and the MC for preoperative planning of M3 extraction surgery to avoid surgical complications. This research could be a cornerstone for the automatic and accurate classification of more diverse relationships between the M3 and the MC.

## Data Availability

The datasets generated and/or analyzed during the current study are not publicly available due to the restriction by the IRB of Seoul National University Dental Hospital to protect patient privacy but are available from the corresponding author on reasonable request. Please contact the corresponding author for any commercial implementation of our research.
